# The Judge's Summing-Up

**Published:** 1916-03-18

**Authors:** 


**MACMILLAN v. NURSING PRESS—(continued).**

### The Judge's Summing-up.

(*Verbatim Report.*)

Mr. Justice Ridley: Gentlemen of the Jury,—This action is brought upon a publication which certainly would be libellous unless you think it is a matter of fair comment upon a matter of public interest. The defendants in such cases have a right to comment as they think proper upon matters of public interest, so long as it is done fairly in the opinion of the jury who have to try the case.

Now, I think this was a matter certainly of public interest, and I believe it is right to say that. The questions such a paper would deal with, I suppose, would be questions of the management of nursing institutions, and among them would be the question of whether nurses ought to be registered or not. I will assume that that would be a question of public interest upon which the defendant has a right to comment, so long as she did it in a fair spirit and in a proper frame of mind. That is to say, first of all the comment itself is to be limited within what you think fair proportions; it has properly and fairly to bear upon what has been done and what is the subject of discussion. And, secondly, even if it does keep within the limits which are right, you have to ask yourselves whether it was said fairly, because you may, whilst you comment fairly upon the subject which is to be determined, you may all the time have got this in your mind—that it is not the subject you want to be at, but it is the person behind that you may have a fancy to be dealing with, while all the time you are pretending to be dealing with the subject. I do not say that this is a case of that description, but I say that is the way in which it has to be looked at.

The case referred to by Mr. Dickens was a case in which *Punch*, the comic journal, was sued for libel in respect of a criticism that had been written in reviewing a book, and the same subject arose there. It went against the publication in that instance, because the Court of Appeal laid down that doctrine. They said this among other things, that proof of malice may take criticism, which *primâ facie* is fair, outside the rule of fair comment, just as it takes a communication which *primâ facie* is privileged outside of privilege.

So that here you have got to ask yourselves not only whether this is fair comment, but whether it is made without malice, and whether, from a fair and proper mind, they are making comments upon the subject dealt with.

### One Great Advantage.

Now, gentlemen, you have had the great advantage in this case of getting all the facts from the plaintiff and her two witnesses, and also of having heard the defendant put her own case forward. And you also have had the advantage of Mr. Macnaghten's way of putting the case before you, than which, if I may be allowed to say so, I do not think anything could be better. Certainly it was concise, very clear, and, if he did not boast of eloquence, at all events one could understand what his point was and we have time to think of it. Now, he says that this is a good and a sound criticism that this journal, the *Nursing Times*, was carrying on. But was it?

### What Mrs. Bedford Fenwick Thought.

The criticism does seem, according to the lady's own account of it—and I do not think I am doing her a wrong—she thought of nothing with regard to nurses but the registration of those nurses. I do not believe I am doing her an injustice when I say that. She stepped into the box and she said, "Yes, that is my subject; that is what I hold to. It is a subject of the greatest importance. We were very nearly in the House of Commons Committee, and almost in the House of Lords Committee, upon this subject." Well, now, I do not think it was of such importance. I mention this, and make these remarks, because when I began what I had to say to you I endeavoured to define what was the subject of her criticism in this case, in order to see whether it was a fair matter to be so criticised.

### Was it Fair Criticism?

She began by saying—let us assume she was right—she began by saying, "That is the point I have got to make." Now, how far does the libel which we have got to inquire into to-day carry out the notion that it was a fair criticism upon the subject of the management of nursing institutions, and particularly upon the question whether nurses ought to be registered or not. Now let us look at it. I shall not be very long—I am not going to weary you by reading the whole of it—but it first of all begins by saying: "We have now in our possession documentary evidence that tms publication. the *Nursing Times*"—that is Messrs. Macmillan's publication—"is edited by 'Fräulein Bulau,' the untrained lady of German parentage, who, under an assumed name, has been resident in England for some years, who was hurriedly naturalised as a British subject three months after the declaration of war, and who has done us the honour of attempting to control our professional affairs in England—of course, for an adequate financial consideration. This significant fact having been established, we may now proceed to invite replies to the following questions from all and sundry whom they may concern." Gentlemen, first of all, I do not deny that in the course of the criticisms made upon nursing institutions it would be quite fair to state the facts, if you state them correctly, so far as they bear upon the nationality of the person who is editing the paper about which she is writing. Not that it bears directly upon the subject, but I think it would be impossible to exclude it. Therefore, if she had correctly stated what the facts are relating to the plaintiff, one would have had no right to complain of it. But let us see what it was that she said. "Fräulein Bulau," to begin with. Now, you have heard that her name is now "Bulan." You have heard the truth, that she has done her best to become an English subject. And are we going to repudiate and thrust back those who do? She had done her best to become a British subject, and because her father's naturalisation certificate granted in New Zealand did not cover her whilst she was in England in 1914, she was obliged to apply to the Home Office and get a fresh certificate. That was the fact. The father had been naturalised in New Zealand, and he and she alike thought the certificate covered their nationality and would make them British subjects so long as they were anywhere within the Dominions. But it seems it was not so. Now, that was the truth of the matter, and, so long as there is no evidence to the contrary, you must take it that all the predilections of the family are in favour of England—they do not even use the German language—and that she has altered the name

"Bulau" to "Bulan" for the reason that was given to you, although the defendant says she does not believe it. There it is. Well, that being so, is this a fair description—"Fräulein Bulau"? Why "Fräulein Bulau," when her name was "Bulan"? Then it goes on, "An untrained lady of German origin." Oh, that is true; she was not trained as a nurse, and she was of German parentage. But what is next? ". . . who, under an assumed name." Gentlemen, is that a fair criticism? Have you got your facts right? Your facts are wrong. It was not an "assumed" name, if you are going to think, as I suppose you will, that an assumed name carries this idea with it, that for some reasons into which it is better not to inquire the real name has been dropped. ". . . under an assumed name has been resident in England for some years, who was hurriedly naturalised as a British subject three months after the declaration of war." Gentlemen, that is not true. That is not a true description of what took place, and one of the rules that we have accepted is that before you go to your criticism on a matter of public interest you must get it right. I listened to the lady in the box, and not a word of explanation was she able to give. This was not a "hurried naturalisation as a British subject." The truth was that they all regarded themselves as naturalised before, and it was only on the discovery, by an application to the Home Office, that it was necessary to have a fresh certificate that it was done.

Well, now, gentlemen, are we going to say that that is a fair criticism of what had taken place about the parentage, about the extraction, about the naturalisation of the plaintiff Bulan, or not? That is for you. But if it is not, it is sufficient in itself to carry this verdict against the defendant, because in that case it is most clearly a libellous statement—most clearly, unless it falls within the category that I have mentioned to you.

### The Questions taken Seriatim.

And now for the questions. First of all, "To the Secretary of State for War,—Is the Matron-in-Chief of the Territorial Force Nursing Service"—that is Miss Browne—"still collaborating with Fräulein Bulau"—there you have it again, "Fräulein Bulau"—"in the production of the *Nursing Times*, and, as heretofore, is she still attending at its office in a subordinate position, and therefore under the control of its German editor?" Insinuating, you see, that Miss Browne, who was in the employment of the War Office, and Matron-in-Chief of the Territorial Force Nursing Service, was under the control of a person called Fräulein Bulau, who had been of German parentage, and who had been "hurriedly" naturalised, as stated in the previous paragraph, and therefore presumably of German influence, and therefore a matter that the Secretary of State, I suppose, had to inquire into was whether this "Fräulein Bulau" was managing and controlling the mind and the disposition of Miss Browne, who was in the employment of the War Office. Gentlemen, that also, so far as the expression "subordinate" goes, is untrue. She was not subordinate at all. You have heard the description of the position that Dr. Rolleston and Miss Browne occupied, and it is not true that they were in a "subordinate" position. She says so, and seems to think that is sufficient, but it is not true.

Then: "To sundry matrons pushing the publication. How about the purity of your professional ethics and patriotism when you secretly associate yourself with Fräulein Bulau? Why do you cover an unprofessional alien who for some reason was in England under an assumed name?" Gentlemen, here we have the same thing. The suggestion is that the matrons who support the publication—the purchasers of this journal—are associating themselves with a lady who is an "unprofessional alien," and who, for some reason or another, nobody knows what, is under an "assumed name" in England. The same thing, I think, over again.

"To the Queen's Nurses,—When your Penny Pension Scheme started, for advertising purposes"—well, that is pretty plain to begin with. I do not know whether you will find that is proved. It does seem that this scheme for pensions for the Queen's Nurses was not a mere advertising scheme, but that it was carried to this point—that some £1,400 was collected, which formed a pension fund for these ladies who are called "Queen's Nurses," a particular class of nurses. That seems to be true, and it is not denied by the defendant. "When your Penny Pension Scheme started, for advertising purposes, from the office of the *Nursing Times*, did you know the hon. secretary of the fund was Fräulein Bulau, and not Miss Bulan? Now that you do know, are you content to be patronised by her?" Gentlemen, why not? Because, of course, it is suggested she was of German sympathies—of sympathies with the nation with whom we are now at war. That is what it means. "Are you content to be patronised by her?" There is no other point, it seems to me, in that sentence. And is it true? Is there a word of truth in it? You have heard the story of the defendant, and you have heard from the plaintiff the history of this family, and you have heard it also, so far as it can be given, by Sir Frederick Macmillan; and do you think there is a word of truth in the suggestion that they ought to be shy, ought to be afraid, ought to avoid, being patronised by her?

And now we come to the last question, to the directors of Macmillan and Co.: "As your publication, the *Nursing Times*, has no ethical significance"—what in the world that means I do not know, but I suppose it has got some meaning. I have heard it said that the Press has no morals; whether that is another phrase having the same meaning I do not know, but "as your publication" (that, of course, is as distinguished from ours, the defendants', publication) "has no ethical significance" must mean that the defendants' has, and I do not see what evidence there can be of that in what the defendant has said to us about her paper. But let that pass. "As your publication, the *Nursing Times*, has no ethical significance, as it has always been edited by an untrained alien, and is, therefore, merely a commercial speculation"—now what a reason, gentlemen—"for how long do you intend to outrage the patriotic feelings of British nurses by employing German control?" Gentlemen, that is the same thing; and so it is with the final question, which I really do not think I need read.

Now, of course, if all these things were to occur separately, if they were not so clear, and were not contained in a bundle of questions one after the other, the case would not be so plain as I venture to think it will be in your opinion to-day. If there was but one question which did suggest that it was improper and undesirable that a person of German sympathies, intending to carry them out and to act contrary to the interests of this country, should be the editor of the paper, you might hesitate over it; you might think, "Oh, no; it is no more than the ordinary feeling that everyone is affected with. After all, I would sooner have a British subject working for me than I would have a German subject, however much he has been naturalised, and whatever his sympathies may be." But, gentlemen, this is repeated in every paragraph, one after the other, and it is repeated in that way without the facts being true, as it appears now, upon the pretext that it is a subject of national importance which gives a privilege to those who fairly discuss it.

The Position of the Press.

Well, it is so, and I ought to add, perhaps, to what I said at the beginning, not merely because it is a question about the nurses, but because I do think it is a matter of importance that we should know whether people who are managing the Press, and who are managing our institutions, are really to be trusted in this time of war or not. That is another reason why she has every right to criticise this matter, so long as she does it fairly, without malice. But when you have heard all the truth about this matter, not only as to the naturalisation of the plaintiff, but as to the management of the newspaper, what do you say?

Another Point of View.

Another point of view is, Was this done once and for all? Is it one attack which the plaintiff can single out and say, "Here, that is a libel"? But, gentlemen, I think if I were to take you in detail through the extracts which have been taken by the plaintiffs out of the defendants' journal, the *British Journal of Nursing*, you will find there has been a systematic attack from the beginning. It began before the poor thing—I mean the just-born *Nursing Times*—had come into existence. It was on April 1, 1905; it was just going to be issued; it had not even come out when the first assault was committed upon it, because it says that the preliminary circular about this new journal makes it perfectly clear that it is a purely commercial concern, and that the well-being of nurses, and the betterment of their financial and professional position, is to be left severely alone—an almost incomprehensible sentence, unless you think this, that as the new journal was going to leave the question of registration alone, therefore the argument is that it can do no good whatever to either nurses or the betterment of their position, and therefore it is useless. That was the sort of argument that was started; at the very beginning there was this attack.

Now, gentlemen, I have very little more to say about it. The matter is entirely one for you to determine. If you take the view that these criticisms were not dictated by a fair measure of what had taken place from knowledge of the events, and that there were other reasons—that is to say, of a malicious nature—a malicious nature as against the plaintiff—which actuated the defendant, it will be a question of damages. That is a matter entirely for you. I do not suppose that pecuniary damages can be shown to accrue either to Miss Bulan or to the owners of the magazine, but at the same time I should have thought you would have come to the conclusion that substantial damages are due from a defendant who in this sort of way—if you find it to be libellous—makes an attack upon a newspaper which is bound to bring it into contempt amongst those who are patriots, and have any wish that their country may succeed in this war. Therefore, gentlemen, if you find for the plaintiff, I think you may find, within reasonable limits, what would be a substantial sum. And you are only to find one sum; you are not to find two sums, although there are two plaintiffs.

The jury then found for the plaintiffs, as recorded above.

**Note.—For want of space we have had to hold over some of the full report of this trial until next week.**

